# Incidence Rate Trends of Breast Cancer Overall and by Molecular Subtype by Race and Ethnicity and Age

**DOI:** 10.1001/jamanetworkopen.2024.56142

**Published:** 2025-01-24

**Authors:** Noelani H. Y. Li, Christopher I. Li

**Affiliations:** 1 Lakeside School, Seattle, Washington; 2 Division of Public Health Sciences, Fred Hutchinson Cancer Center, Seattle, Washington

## Abstract

**Importance:**

Black and Hispanic women in the US experience higher incidence rates of aggressive molecular subtypes of breast cancer, including triple-negative disease. However, how these rates are changing, particularly across different age groups, has not been well documented.

**Objective:**

To assess changes in overall and subtype-specific breast cancer incidence rates in the US by age and race and ethnicity.

**Design, Setting, and Participants:**

This cohort study used Surveillance, Epidemiology, and End Results program cancer registry data from 22 US cancer registries on 1 123 658 females who received a diagnosis of invasive breast cancer from 2010 to 2019. Statistical analysis was conducted from August 2023 to October 2024.

**Exposures:**

Age and race and ethnicity.

**Main Outcomes and Measures:**

Age-adjusted incidence rates of invasive breast cancer overall and across the 4 major molecular subtypes by age and by race and ethnicity, as well as their associated annual percentage changes using Joinpoint Trend Analysis software.

**Results:**

Of the 1 123 658 participants in the study, 219 112 (19.5%) were younger than 50 years, 409 257 (36.4%) were aged 50 to 64 years, and 495 289 (44.1%) were 65 years or older. A total of 141 703 participants (12.6%) were Hispanic, 3253 (0.3%) were non-Hispanic American Indian or Alaska Native, 78 306 (7.0%) were non-Hispanic Asian or Pacific Islander, 124 560 (11.1%) were non-Hispanic Black, 769 043 (68.4%) were non-Hispanic White, and 6793 participants (0.6%) had an unknown race and/or ethnicity. Overall, breast cancer incidence rates increased 0.5% per year from 2010 to 2019. Variation by race and ethnicity was observed, with increases of 1.4% per year among Hispanic females, 1.9% per year among non-Hispanic American Indian or Alaska Native females, and 2.1% per year among non-Hispanic Asian or Pacific Islander females, while rates increased only 0.8% per year among non-Hispanic Black females and 0.5% per year among non-Hispanic White females. In subtype analyses, increases of the greatest magnitude in recent years were observed in the incidence rates of triple-negative breast cancer per year among participants aged 65 years or older (Hispanic females, 2.3%; non-Hispanic Asian or Pacific Islander females, 5.5%; and non-Hispanic Black females, 4.3%), while remaining unchanged among non-Hispanic White females.

**Conclusions and Relevance:**

In this cohort study of 1 123 658 females with breast cancer over the 10-year period from 2010 to 2019, there were substantial differences in trends in the incidence rates of breast cancer overall and by subtype across different racial and ethnic groups. Further research is needed to understand the factors associated with these trends.

## Key Points

**Question:**

How have incidence rates of breast cancer changed according to molecular subtype, age, and race and ethnicity in the US from 2010 to 2019?

**Findings:**

This cohort study found that, although breast cancer rates overall continue to increase 0.5% per year in a large cohort of US women, disproportionately higher increases have been observed among Hispanic, non-Hispanic American Indian or Alaska Native, and non-Hispanic Asian or Pacific Islander females and variation was also observed by molecular subtype.

**Meaning:**

This study’s findings on incidence rates of breast cancer among racial and ethnic groups suggests that understanding factors associated with these trends, particularly among the more aggressive and lethal subtypes of breast cancer, is of public health importance.

## Teaser

This cohort study assesses changes from 2010 to 2019 in overall and subtype-specific breast cancer incidence rates in the US by age and race and ethnicity.

## Introduction

Among women, breast cancer is the most commonly diagnosed cancer and the leading cause of cancer-related death globally.^1^ In the US over the past decade from 2010 to 2019, breast cancer incidence rates have increased 0.5% annually.^2^ However, breast cancer is a heterogeneous disease consisting of 4 main subtypes defined by joint hormone receptor (HR) and ERBB2 status: HR-positive and ERBB2-negative, HR-negative and ERBB2-negative, HR-positive and ERBB2-positive, and HR-negative and ERBB2-positive. Incidence rates vary by subtype. Hormone receptor–positive and ERBB2-negative breast cancer is the most common subtype, while HR-negative and ERBB2-positive breast cancer is the rarest.^3^ Subtypes also vary in their clinical aggressiveness and treatability, with HR-negative and ERBB2-negative breast cancer being the most lethal and difficult to treat.^4^

In addition, overall and subtype-specific incidence rates vary considerably by race and ethnicity and age.^5^ For example, incidence rates of estrogen receptor (ER)-negative breast cancers are higher among African American women compared with women of other races and ethnicities.^6^ A recent Surveillance, Epidemiology, and End Results (SEER) program–based study using data from 2010 to 2016 showed that incidence rates of HR-positive and ERBB2-negative breast cancer have increased among non-Hispanic White (hereafter, *White*) women (annual percentage change [APC] from 2010 to 2014, 2.3%) of all ages, non-Hispanic Asian or Pacific Islander (hereafter, *Asian or Pacific Islander*) women aged 40 to 54 years (APC from 2010 to 2016, 2.5%), and non-Hispanic Black (hereafter, *Black*) women aged 55 to 69 years (APC from 2010 to 2012, 4.9%).^7^ However, this study was limited to data from 2010 to 2016, assessed only linear trends in rates over this time period, and did not include non-Hispanic American Indian or Alaska Native (hereafter, *American Indian or Alaska Native*) women.

A current gap in knowledge is how breast cancer subtype-specific incidence rates vary by race and ethnicity and age over the past decade from 2010 to 2019 and whether changes in these rates are accelerating or decelerating from what has been previously observed. Using cancer registry data from the SEER program, this study describes recent trends in the incidence rates of different breast cancer subtypes stratified by race and ethnicity and age.

## Methods

### Study Population

This cohort study used data from the 22 SEER cancer registries included in the SEER Explorer Application.^8^ These registries capture cancer incidence rates in California, Connecticut, Georgia, Hawaii, Idaho, Iowa, Illinois, Kentucky, Louisiana, Massachusetts, New Jersey, New Mexico, New York, Texas, and Utah. In addition, the registry covering the 13-county Seattle-Puget Sound region of western Washington state and the Alaska Native Tumor Registry were included. Together, these registries cover 47.9% of the total US population.^9^ Our study population was limited to females of all ages who received a diagnosis of invasive breast cancer identified through the 22 cancer registries included regardless of prior cancer history. This study focused primarily on the 1 123 658 breast cancer cases diagnosed from 2010 to 2019. The starting year for subtype specific analyses was 2010 because it is the earliest year data on both HR and ERBB2 status are available. Patients were defined as HR positive if their tumor was either ER positive or progesterone receptor (PR) positive and as HR negative if their tumor was both ER negative and PR negative. Data from 2020 onward were not included because of the well-documented effect of the COVID-19 pandemic on SEER cancer incidence data.^10^ For overall breast cancer incidence rates, the data included span 2000 to 2019 to provide additional historical context. Data on age, race and ethnicity, HR status, ERBB2 status, and other tumor characteristics are abstracted from medical records and pathology reports by SEER registry staff members using standardized coding approaches.^11^ Because the data used in this study were all publicly available and deidentified, this study was exempt from human participants review and consent was waived. The Strengthening the Reporting of Observational Studies in Epidemiology (STROBE) reporting guideline for cohort studies was followed.

### Statistical Analysis

Statistical analysis was conducted from August 2023 to October 2024. Age-adjusted incidence rates per 100 000 overall and by subtype (HR-positive and ERBB2-negative, HR-positive and ERBB2-positive, HR-negative and ERBB2-positive, and HR-negative and ERBB2-negative), age (<50, 50-64, and ≥65 years), and race and ethnicity (American Indian or Alaska Native, non-Hispanic Asian or Pacific Islander, Black, Hispanic [any race], and White) were calculated using SEER*Explorer software using data from the US Census Bureau as the denominators. The racial and ethnic groupings used were those defined by the SEER program.^12^ Annual percentage changes were calculated using the Joinpoint Trend Analysis software, version 5.1 (National Cancer Institute) for all incidence rate trends evaluated using the default settings that enable changes in the direction and/or magnitude of incidence rate trends to be quantified over a given time period. Two-sided *P* values for trend using α = .05 were used to define trends that were statistically significant.

## Results

Of the total of 1 123 658 participants with invasive female breast cancer who received a diagnosis from 2010 to 2019 across the 22 SEER cancer registries included in this study, 219 112 (19.5%) were younger than 50 years, 409 257 (36.4%) were aged 50 to 64 years, and 495 289 (44.1%) were 65 years or older (eTable in Supplement 1). In addition, 3253 participants (0.3%) were American Indian or Alaska Native, 78 306 (7.0%) were Asian or Pacific Islander, 124 560 (11.1%) were Black, 141 703 (12.6%) were Hispanic (any race), and 769 043 (68.4%) were White. Only 6793 females (0.6%) included had an unknown race and/or ethnicity; thus, exclusion of this small proportion of cases is unlikely to have a meaningful association with our results. According to breast cancer subtype, 749 790 (73.9%) were HR-positive and ERBB2-negative, 114 249 (11.3%) were HR-negative and ERBB2-negative, 106 139 (10.5%) were HR-positive and ERBB2-positive, and 44 799 (4.4% were HR-negative and ERBB2-positive. As expected, the proportion of cases that were HR-positive and ERBB2-negative increased with age, and compared with other racial and ethnic groups, Black females had the highest proportion of HR-negative and ERBB2-negative breast cancer (18.6% [23 213 of 124 560]) followed by Hispanic females (11.3% [16 036 of 141 703]). Also, the proportion of patients with an unknown breast cancer subtype decreased steadily from 15.4% (15 490 of 100 748) in 2010 to 6.2% (7770 of 125 141) in 2019. Differences in the rates or magnitudes of these decreases by race and ethnicity could affect the interpretation of our results. To address this possibility, we evaluated race and ethnicity–specific proportions of unknown breast cancer subtype by year and found that the proportions of patients with an unknown breast cancer subtype did not vary appreciably by race and ethnicity. Specifically, compared with proportions of White patients (our largest racial and ethnic group) with an unknown subtype in individual years from 2010 to 2019, the proportions of Hispanic patients with an unknown breast cancer subtype were within 1.9% to 3.5% of the White proportions across each year, the proportions among American Indian or Alaska Native patients were within 0.0% to 5.7%, the proportions among Asian or Pacific Islander patients were within 0.1% to 1.5%, and the proportions among Black patients were within 0.9% to 2.8%. These results suggest that the decreases in the proportions of patients with an unknown breast cancer subtype were consistent across all racial and ethnic groups included and are unlikely to have a meaningful association with our findings.

**Figure 1.**
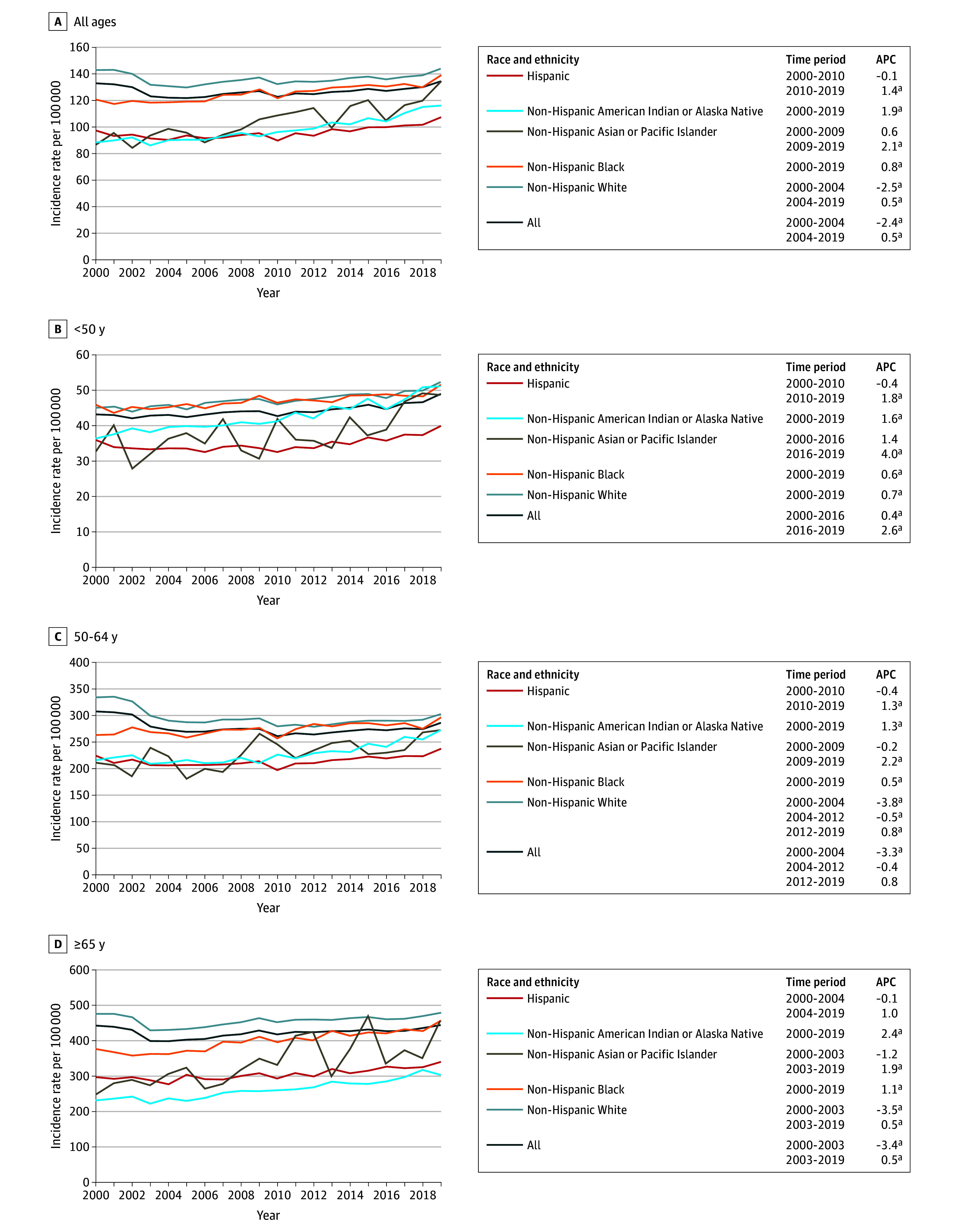
Overall Age-Adjusted Female Breast Cancer Incidence Rates (Per 100 000) and Annual Percentage Changes (APCs) From 2000 to 2019 by Race and Ethnicity Across Different Age Groups ^a^*P* < .05.

For females of all ages and all races and ethnicities, overall breast cancer incidence rates increased 0.5% per year from 2004 to 2019 (Figure 1). Variation by race and ethnicity was observed, with increases of 1.4% per year (from 2010 to 2019) among Hispanic females, 1.9% per year (from 2000 to 2019) among American Indian or Alaska Native females, and 2.1% per year (from 2009 to 2019) among Asian or Pacific Islander females, while rates increased only 0.8% per year (from 2000 to 2019) among Black females and 0.5% per year (from 2004 to 2019) among White females. Generally similar patterns were observed when rates were stratified by age. Among those younger than 50 years, rates increased 0.6% per year among Black females and 0.7% per year among White females, but increased at rates more than double this APC among American Indian or Alaska Native, Asian or Pacific Islander, and Hispanic females.

**Figure 2.**
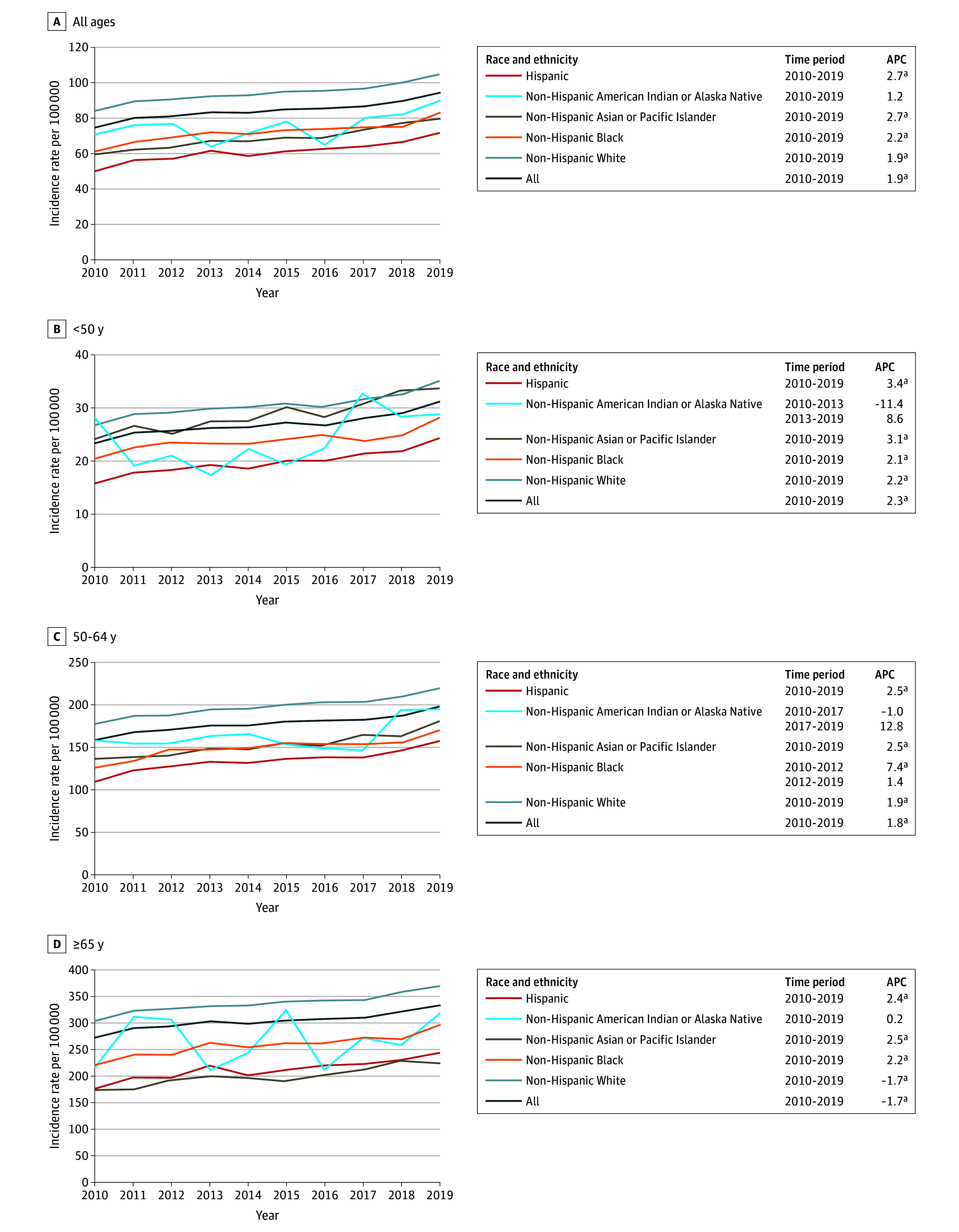
Age-Adjusted Incidence Rates (Per 100 000) and Annual Percentage Changes (APCs) for Hormone Receptor–Positive and ERBB2-Negative Female Breast Cancer From 2010 to 2019 by Race and Ethnicity Across Different Age Groups ^a^*P* < .05.

Overall, the incidence rate of HR-positive and ERBB2-negative breast cancer for all races and all ages increased by 1.9% per year from 2010 to 2019 (Figure 2). However, rates increased more rapidly among Hispanic and Asian or Pacific Islander females (by 2.7% per year for each group). Similar patterns were observed when results were stratified by age, with Hispanic and Asian or Pacific Islander females experiencing greater increases compared with other racial and ethnic groups. Due to smaller sample sizes, there was greater fluctuation in the year-to-year incidence rates among American Indian or Alaska Native females.

**Figure 3.**
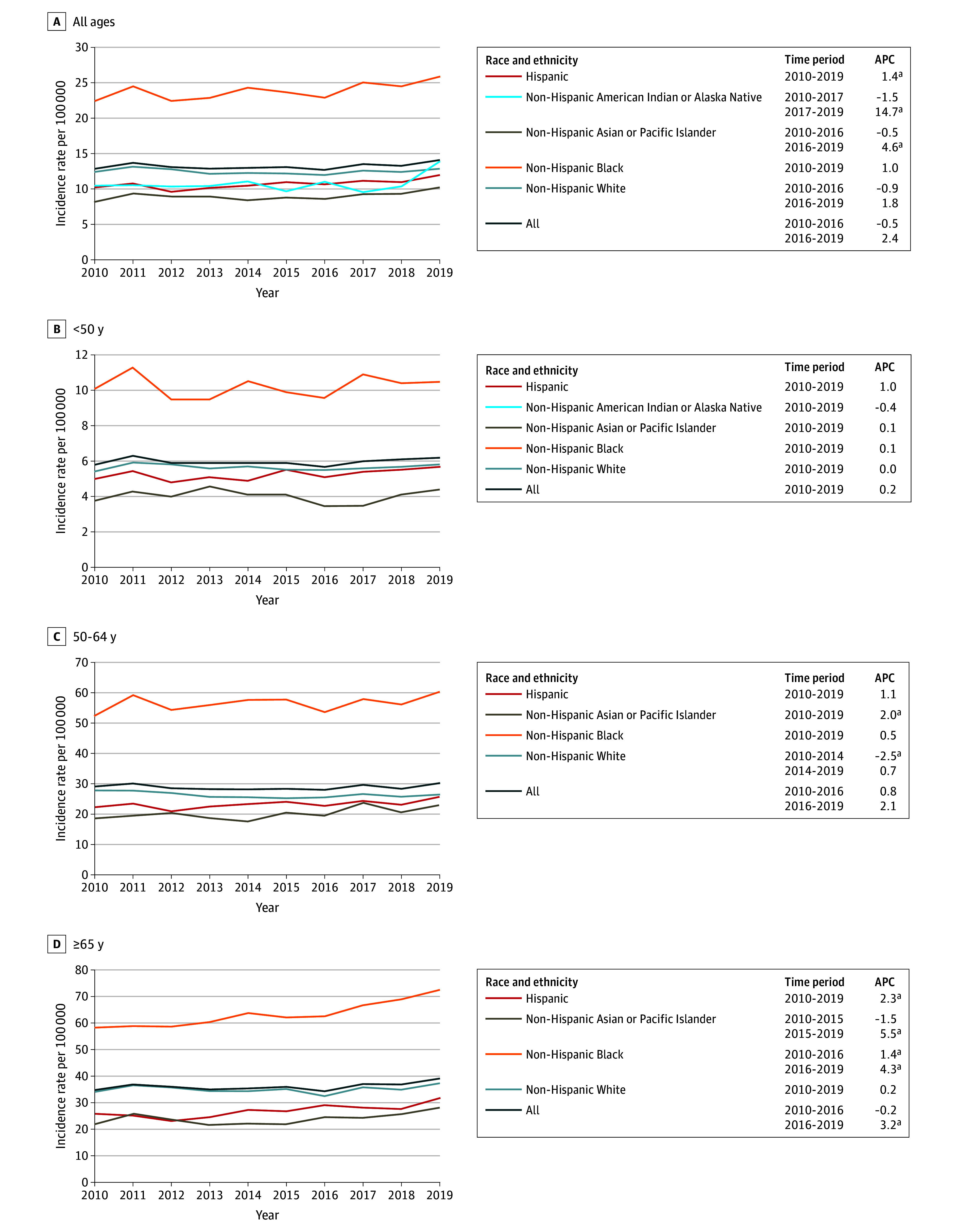
Age-Adjusted Incidence Rates (Per 100 000) and Annual Percentage Changes (APCs) for Hormone Receptor–Negative and ERBB2-Negative Female Breast Cancer From 2010 to 2019 by Race and Ethnicity Across Different Age Groups Age-specific incidence rates of non-Hispanic American Indian or Alaska Native females were based on too few cases for incidence rates to be calculated. ^a^*P* < .05.

Among females of all ages and races and ethnicities, there was no statistically significant change in the incidence rates of HR-negative and ERBB2-negative (triple-negative) breast cancer (Figure 3), but considerable variation by age and race and ethnicity was observed. The only group that experienced no changes in incidence rates, with the exception of a short-term decrease in rates only among females aged 50 to 64 years from 2010 to 2014, were White females. In contrast, increases were observed among Asian or Pacific Islander women who were 50 to 64 years of age (2.0% increase per year from 2010 to 2019) and 65 years or older (5.5% increase per year from 2015 to 2019). In addition, among those aged 65 years or older, increases were observed among Hispanic females (2.3% increase per year from 2010 to 2019) and Black females (1.4% increase per year from 2010 to 2016 and 4.3% increase per year from 2016 to 2019). In contrast, there were no changes in rates among females younger than 50 years across all racial and ethnic groups. Although for the other types of breast cancer incidence rates showed minimal variation across different racial and ethnic groups, for HR-negative and ERBB2-negative breast cancer Black females had rates that were approximately double those of the other racial and ethnic groups; this finding was consistent across all age groups.

**Figure 4.**
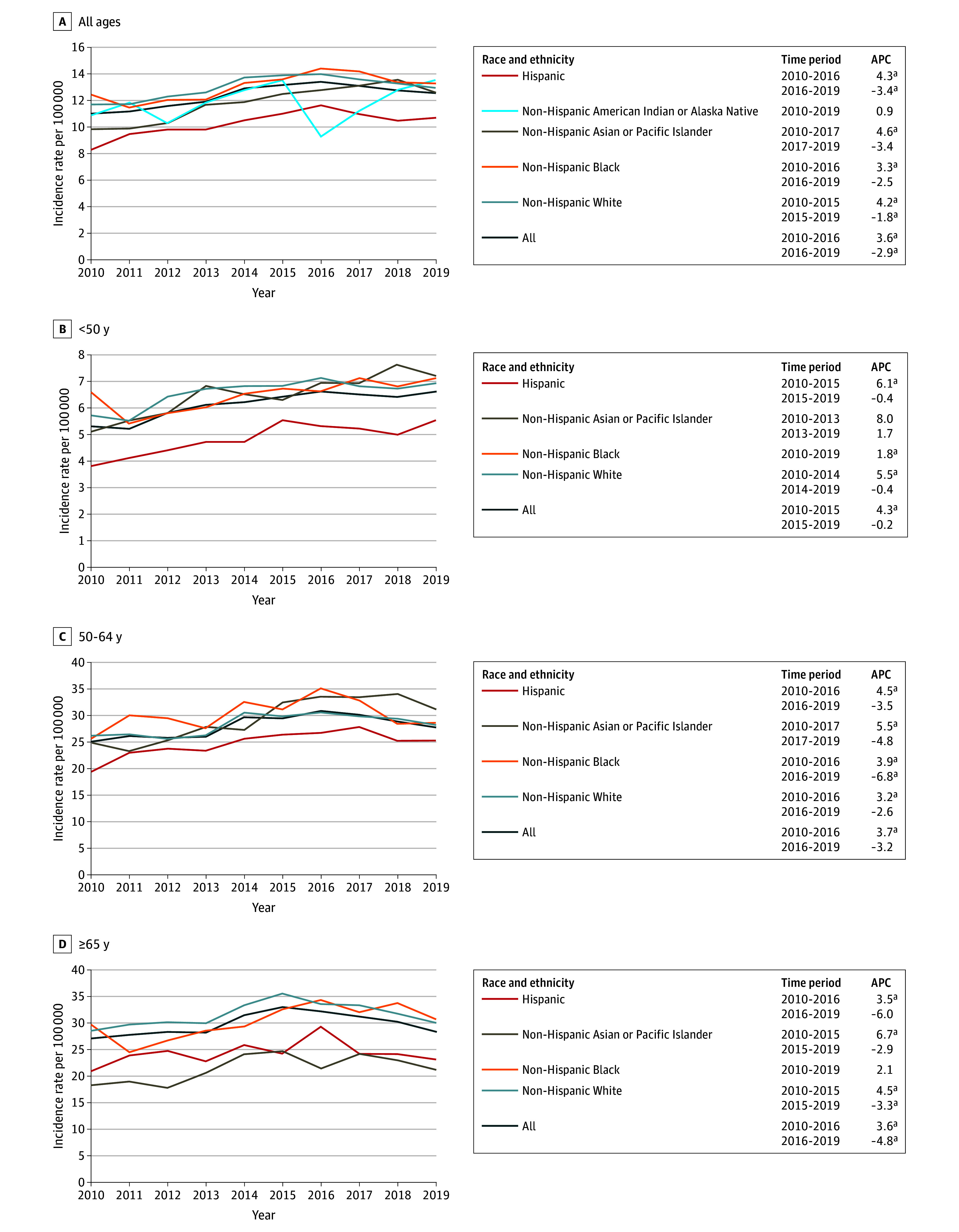
Age-Adjusted Incidence Rates (Per 100 000) and Annual Percentage Changes (APCs) for Hormone Receptor–Positive and ERBB2-Positive Female Breast Cancer From 2010 to 2019 by Race and Ethnicity Across Different Age Groups Age-specific incidence rates of non-Hispanic American Indian or Alaska Native females were based on too few cases for incidence rates to be calculated. ^a^*P* < .05.

The overall incidence rate of HR-positive and ERBB2-positive breast cancer increased 3.6% per year from 2010 to 2016, but then decreased by 2.9% per year from 2016 to 2019, with a similar pattern observed across different age groups (Figure 4). However, some variation was observed by race and ethnicity. Rates increased from 2010 to 2019 among Black females who were younger than 50 years (increasing 1.8% per year) and those who were 65 years or older (increasing 2.1% per year, although this trend was not statistically significant).

**Figure 5.**
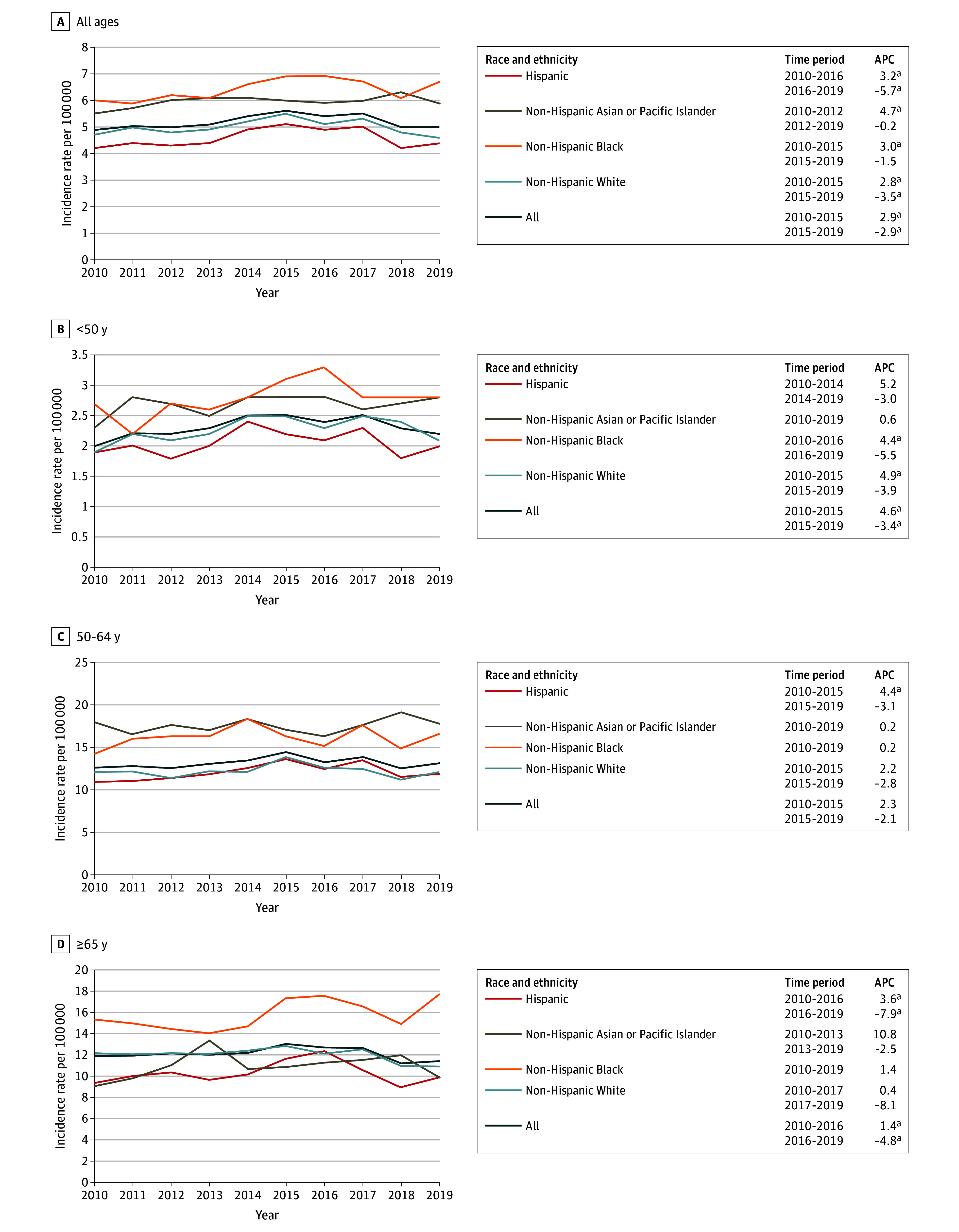
Age-Adjusted Incidence Rates (Per 100 000) and Annual Percentge Changes (APCs) for Hormone Receptor–Negative and ERBB2-Positive Female Breast Cancer From 2010 to 2019 by Race and Ethnicity Across Different Age Groups Overall and age-specific incidence rates of non-Hispanic American Indian or Alaska Native females were based on too few cases for incidence rates to be calculated. ^a^*P* < .05.

Overall, incidence rates of HR-negative and ERBB2-positive breast cancer increased 2.9% per year from 2010 to 2015 and then decreased 2.9% per year from 2015 to 2019, with a similar pattern observed across all age groups (Figure 5). This pattern was generally consistent across racial and ethnic groups, with the exception of Asian or Pacific Islander females younger than 50 years and 50 to 64 years of age and Black females 50 to 64 years of age and 65 years or older whose incidence rates of HR-negative and ERBB2-positive breast cancer remained statistically unchanged from 2010 to 2019.

## Discussion

We observed marked differences in incidence rate trends of different subtypes of breast cancer by age and race and ethnicity from 2010 to 2019. Consistent with prior published work,^2^ we observed an overall 0.5% per year increase in breast cancer incidence rates in the US since 2004, but we found that from 2010 to 2019 rates have increased more quickly among Hispanic females (1.4% per year since 2010) and Asian or Pacific Islander females (2.1% per year since 2009). A prior study using national data from the North American Association of Central Cancer Registries from 1999 to 2017 evaluated only 3 racial and ethnic groups (Black, Hispanic, and White), where they observed similar APCs across all 3 groups (Black, 0.5% per year from 2008 to 2017; White, 0.4% per year from 2004 to 2017; and Hispanic, 0.6% per year from 2004 to 2017).^13^ Although our results are not directly comparable with those of that study given that they are based on different populations, our findings suggest an acceleration of the increasing incidence of breast cancer among Asian or Pacific Islander and Hispanic women over more recent years. Patterns were generally consistent across different age groups. For example, with respect to young-onset breast cancer (incidence rates among those <50 years), while rates increased 0.6% per year among Black females and 0.7% per year among White females from 2000 to 2019, they increased at rates more than double this APC among American Indian or Alaska Native, Asian or Pacific Islander, and Hispanic females. Similar patterns were also observed in rates among females 50 to 64 years of age and 65 years or older. Given the descriptive nature of this study, we cannot infer what factors are associated with these trends. Differences in the use of breast cancer screening could have some association with trends, although screening rates among women 50 to 74 years of age are lowest among American Indian or Alaska Native women (59%) and Asian women (67%), highest among Black women (82%), and in-between for Hispanic women (74%) and White women (76%), and overall breast cancer screening rates in the US have held essentially constant since 2000.^14^

Providing further insights into the potential factors associated with these trends are our results by molecular subtype. Beginning with the HR-negative and ERBB2-negative subtype, commonly referred to as *triple-negative breast cancer*, our results indicate that rates have held essentially constant across all racial and ethnic groups among women younger than 50 years, but marked differences were observed among women 65 years or older. Although rates have been unchanged among White females in this age group, among Black females they increased 1.4% per year from 2010 to 2016 and to 4.3% per year from 2016 to 2019. Hispanic women in this age group experienced a 2.3% increase per year from 2010 to 2019 and Asian or Pacific Islander females experienced a 5.5% increase per year from 2015 to 2019. On an absolute scale, Black women continue to have substantially higher incidence rates of triple-negative breast cancer compared with all other racial and ethnic groups, with rates that are approximately double those of all other groups across all ages. Although this observation has been previously reported,^3^ ours is the first study to our knowledge to note the increasing incidence of triple-negative breast cancer among certain racial and ethnic groups in older women. A study reporting on SEER data from 2010-2016^7^ did not report any statistically significant increases in the incidence rates of triple-negative breast cancer in any age group within any racial or ethnic group it assessed. This finding is generally consistent with our findings, given that the increases in rates we observed have occurred in more recent years.

In contrast to differences in incidence rate trends of triple-negative breast cancer by race and ethnicity are the more generally consistent trends across racial and ethnic groups for the other breast cancer subtypes. Specifically, rates of HR-positive and ERBB2-negative breast cancer are generally increasing, while in more recent years rates of HR-positive and ERBB2-positive and HR-negative and ERBB2-positive cancers are decreasing. The factors associated with these trends, along with the increase in triple-negative breast cancer among older Asian or Pacific Islander, Black, and Hispanic females while the incidence of triple-negative breast cancer has held entirely flat among White females, are unknown and warrants further investigation to see if these trends continue to persist. Differences in risk factor associations with different breast cancer subtypes could to some extent account for these observations. A recent scoping literature review summarized risk factors by breast cancer subtype across different racial and ethnic groups.^15^ Although it reinforced the established causal heterogeneity that exists by breast cancer subtype, which is the strongest for certain reproductive characteristics, use of exogenous hormones, and body mass index, it also recognized that this literature is based largely on predominantly White populations, and there is a dearth of evidence regarding associations between these risk factors and risk of different breast cancer subtypes among women who are not White. Although certainly further research is warranted, differences in exposure histories to particular breast cancer risk factors by race and ethnicity could account for some of the trends observed. For example, 3 recent review articles have documented that breastfeeding is more strongly inversely associated with risk of triple-negative breast cancer compared with other subtypes.^16,17,18^ Considerable differences in breastfeeding history by race and ethnicity in the US have been reported, with a recent study finding that the proportion of women reporting having initiated breastfeeding at the time of hospital discharge after the birth of their infant was lowest among Black mothers (74.5%) and American Indian or Alaska Native mothers (77.7%), followed by White (85.9%), Hispanic (86.8%), and Asian (90.1%) mothers.^19^ Thus, differences such as these as well as temporal shifts in risk factor profiles across different population subgroups could account for some of the trends we observed.

### Strengths and Limitations

This study has several key strengths. It includes a large nationally representative sample with data from nearly 50% of the US population, enhancing the generalizability of our results to the entire US population. In addition, the data were collected using the rigorous standards of the SEER program, ensuring both high quality and a high level of data completeness.

It is important to acknowledge the limitations of this study. We lacked data on factors such as reproductive factors, anthropometric measures, and other breast cancer risk factors that could be associated with the trends that we observed. In addition, data on race and ethnicity were abstracted from medical records, so there is potential for misclassification. The extent of this misclassification is likely to vary by geographic region, but a study that compared self-reported race and ethnicity with registry-defined race and ethnicity from the Greater Bay Area Cancer Registry found disagreement among 11% of the sample.^20^ It is unknown to what extent this misclassification could be associated with the magnitude or direction of the results reported here. Another limitation is that there are different approaches used to test for ER, PR, and ERBB2 expression across hospitals and laboratories, which could be associated with different patterns of test results. Also, as shown in the eTable in Supplement 1, the proportion of patients with an unknown breast cancer subtype decreased steadily from 15.4% in 2010 to 6.2% in 2019, but as described above no appreciable differences in the rates of these decreases were observed by race and ethnicity. Given these findings and that the magnitude of the differences present is considerably smaller than the magnitudes of the different statistically significant trends in breast cancer incidence rates we observed by race and ethnicity, the association of the missing subtype data with our results is likely to be minimal, and this factor is likely insufficient to account for the differential trends by race and ethnicity observed. Finally, some of the trends observed have been present for relatively short recent periods, so ongoing surveillance of these trends is needed to assess their robustness.

## Conclusions

In this cohort study, we observed key differences in the incidence rates of different subtypes of breast cancer by age and race and ethnicity. Older Asian or Pacific Islander, Black, and Hispanic women are experiencing increases in the incidence of triple-negative breast cancer, which is more aggressive and harder to treat than other subtypes. Further research is needed to understand what factors are associated with these trends, and future work should continue to track how they are changing.

## Notes

Supplement 1. **eTable.** Characteristics of Invasive Female Breast Cancer Cases Diagnosed From 2010-2019

Supplement 2.
Data Sharing Statement
