# Supplementary material for: Incidence Rate Trends of Breast Cancer Overall and by Molecular Subtype by Race and Ethnicity and Age
Source: JAMA Netw Open. 2025 Jan 24;8(1):e2456142. doi: 10.1001/jamanetworkopen.2024.56142 (PMC11762241; doi:10.1001/jamanetworkopen.2024.56142)
Supplement: Supplement 1. — eTable. Characteristics of Invasive Female Breast Cancer Cases Diagnosed From 2010-2019 [file jamanetwopen-e2456142-s001.pdf]

Supplementary Online Content

Li NHY, Li CI. Incidence rate trends of breast cancer overall and by molecular subtype by race and ethnicity and age. *JAMA Netw Open.* 2025;8(1):e2456142. doi:10.1001/jamanetworkopen.2024.56142

eTable. Characteristics of Invasive Female Breast Cancer Cases Diagnosed From 2010-2019

This supplementary material has been provided by the authors to give readers additional information about their work.

© 2025 Li et al. *JAMA Network Open.*

eTable. Characteristics of Invasive Female Breast Cancer Cases Diagnosed from 2010-2019

	All cases	HR+/HER2-		HR-/HER2-		HR+/HER2+		HR-/HER2+		Unknown subtype	
	n=1,123,658	n=749,790		n=114,249		n=106,139		n=44,799		n=108,681	
	n	n	%	n	%	n	%	n	%	n	%
**Age**											
<50	219,112	130,367	59.5%	29,075	13.3%	29,537	13.5%	11,282	5.1%	18,851	8.6%
50-64	409,257	268,993	65.7%	43,329	10.6%	41,900	10.2%	19,527	4.8%	35,508	8.7%
65+	495,289	350,430	70.8%	41,845	8.4%	34,702	7.0%	13,990	2.8%	54,322	11.0%
**Race and ethnicity**											
Hispanic (any race)	141,703	86,894	61.3%	16,036	11.3%	15,431	10.9%	6,918	4.9%	16,424	11.6%
Non-Hispanic American Indian/Alaska Native	3,253	2,166	66.6%	300	9.2%	345	10.6%	134	4.1%	308	9.5%
Non-Hispanic Asian/Pacific Islander	78,306	51,348	65.6%	6,773	8.6%	8,852	11.3%	4,522	5.8%	6,811	8.7%
Non-Hispanic Black	124,560	69,176	55.5%	23,213	18.6%	12,539	10.1%	6,229	5.0%	13,403	10.8%
Non-Hispanic White	769,043	536,288	69.7%	67,390	8.8%	68,389	8.9%	26,713	3.5%	70,263	9.1%
Unknown	6,793	3,918	57.7%	537	7.9%	583	8.6%	283	4.2%	1,472	21.7%
**Diagnosis year**											
2010	100,748	61,876	61.4%	10,464	10.4%	8,931	8.9%	3,987	4.0%	15,490	15.4%
2011	104,508	66,914	64.0%	11,258	10.8%	9,226	8.8%	4,167	4.0%	12,943	12.4%
2012	105,932	69,036	65.2%	10,894	10.3%	9,641	9.1%	4,205	4.0%	12,156	11.5%
2013	108,836	72,049	66.2%	10,895	10.0%	10,002	9.2%	4,339	4.0%	11,551	10.6%
2014	111,306	73,091	65.7%	11,166	10.0%	11,060	9.9%	4,602	4.1%	11,387	10.2%
2015	114,432	76,258	66.6%	11,406	10.0%	11,462	10.0%	4,931	4.3%	10,375	9.1%
2016	114,764	77,719	67.7%	11,228	9.8%	11,770	10.3%	4,721	4.1%	9,326	8.1%
2017	117,748	79,988	67.9%	12,061	10.2%	11,589	9.8%	4,888	4.2%	9,222	7.8%
2018	120,243	83,928	69.8%	12,068	10.0%	11,361	9.4%	4,425	3.7%	8,461	7.0%
2019	125,141	88,931	71.1%	12,809	10.2%	11,097	8.9%	4,534	3.6%	7,770	6.2%

Note: All percentages shown are row percentages.

© 2025 Li et al. *JAMA Network Open.*
