# Supplementary material for: Incidence Rate Trends of Breast Cancer Overall and by Molecular Subtype by Race and Ethnicity and Age
Source: JAMA Netw Open. 2025 Jan 24;8(1):e2456142. doi: 10.1001/jamanetworkopen.2024.56142 (PMC11762241; doi:10.1001/jamanetworkopen.2024.56142)
Supplement: Supplement 2. — Data Sharing Statement [file jamanetwopen-e2456142-s002.pdf]

Data Sharing Statement

Li. Incidence Rate Trends of Breast Cancer Overall and by Molecular Subtype by Race and Ethnicity and Age. *JAMA Netw Open*. Published January 24, 2025.
doi:10.1001/jamanetworkopen.2024.56142

Data
Data available: Yes
Data types: Deidentified participant data, Data (not involving human participants)
How to access data: https://seer.cancer.gov/statistics-network/explorer/application.html
When available: With publication

Supporting Documents
Document types: None

Additional Information
Who can access the data: anyone requesting the data
Types of analyses: any purpose, the data are free and publicly available
Mechanisms of data availability: without investigator support, data are available at:
https://seer.cancer.gov/statistics-network/explorer/application.html
Any additional restrictions: none
